# Dynamic Endothelial Cell Rearrangements Drive Developmental Vessel Regression

**DOI:** 10.1371/journal.pbio.1002125

**Published:** 2015-04-17

**Authors:** Claudio A. Franco, Martin L. Jones, Miguel O. Bernabeu, Ilse Geudens, Thomas Mathivet, Andre Rosa, Felicia M. Lopes, Aida P. Lima, Anan Ragab, Russell T. Collins, Li-Kun Phng, Peter V. Coveney, Holger Gerhardt

**Affiliations:** 1 Vascular Biology Laboratory, London Research Institute—Cancer Research UK, Lincoln’s Inn Laboratories, London, United Kingdom; 2 Instituto de Medicina Molecular, Faculdade de Medicina Universidade de Lisboa, Lisboa, Portugal; 3 Centre for Computational Science, Department of Chemistry, University College London, London, United Kingdom; 4 CoMPLEX, University College London, Physics Building, London, United Kingdom; 5 Usher Institute of Population Health Sciences and Informatics, The University of Edinburgh, No. 9 Edinburgh Bioquarter, Edinburgh, United Kingdom; 6 Vascular Patterning Laboratory, Vesalius Research Center, KU Leuven, Department of Oncology, VIB3, Leuven, Belgium; Duke University Medical Center, UNITED STATES

## Abstract

Patterning of functional blood vessel networks is achieved by pruning of superfluous connections. The cellular and molecular principles of vessel regression are poorly understood. Here we show that regression is mediated by dynamic and polarized migration of endothelial cells, representing anastomosis in reverse. Establishing and analyzing the first axial polarity map of all endothelial cells in a remodeling vascular network, we propose that balanced movement of cells maintains the primitive plexus under low shear conditions in a metastable dynamic state. We predict that flow-induced polarized migration of endothelial cells breaks symmetry and leads to stabilization of high flow/shear segments and regression of adjacent low flow/shear segments.

## Abstract 2

A study of mouse retina and zebrafish vasculature shows how differential blood flow patterns direct the orientation and migration of endothelial cells as a mechanism for stabilizing or pruning individual blood vessel segments.

## Author Summary

The question of how blood vessel networks achieve their branching patterns is key to our understanding of organ formation as well as diseases that involve vascular anomalies. Regression (or pruning) of blood vessel segments is required for functional vascular branching patterns; however, the molecular basis for this is poorly understood. Here we investigate remodeling of vascular networks in the mouse retina and in zebrafish and focus on the cellular components of the endothelium—the cell layer that lines blood vessels. We use high-resolution imaging to map and analyze endothelial cell orientation in relation to blood flow direction during vascular remodeling. We identify sequential steps that characterize blood vessel regression through endothelial cell migration, finding no evidence for predicted endothelial cell death in the retina. Combining endothelial cell mapping with computational modeling of flow-induced shear forces allows a systems-level prediction of endothelial cell migration patterns that drive vascular remodeling. Our work establishes how local differences in blood flow drive endothelial cells to orientate and migrate against the direction of flow. We show that the dynamic and polarized migration of endothelial cells leads to the regression of segments under low flow and the stabilization of segments under high flow. We propose that strong flow functions as an “attractor” for endothelial cells, while poorly perfused vessels are less “attractive,” thereby promoting regression of non-functional vessel segments.

## Introduction

The formation of a functionally perfused and hierarchically branched network of blood vessels is essential for vertebrate development, tissue growth, and organ physiology [1]. Together, vasculogenic vessel assembly and angiogenic sprouting establish the major axial vessels and form a rough draft of a network, which undergoes extensive remodeling to become functional. Also, in the adult, previously quiescent and functional networks can be reactivated, expanded to meet changing metabolic demands, or remodeled, as a consequence of injury or local occlusion. A large number of mouse mutants present defects in vascular remodeling [1,2], yet surprisingly little is known about the cellular principles and the molecular control of remodeling. One critical aspect of remodeling is segment regression, in which previously present connections between two vessel segments are lost. Endothelial cell death has been identified as a major mechanism of programmed regression of the ocular hyaloid vessels [3] and pupillary membrane [4], while in the rat retina, vessel regression occurs without evident cell death [5]. Dynamic imaging has confirmed these distinctions. In the pupillary membrane, network regression is associated with apoptosis-mediated flow restriction [4]. By contrast, in the zebrafish brain, real-time imaging showed that endothelial cells move out of the regressing branch and rarely undergo apoptosis [6,7]. Molecular and physical signals appear to be jointly involved in the process: delta-like ligand 4 (Dll4)/Notch signaling is required for vessel remodeling in the mouse retina, and vessel constriction promotes branch regression [8]. Low or fluctuating flow appears to predetermine branch regression, and enhanced flow protects vessel branches from regression [6]. Our previous work in mouse and zebrafish illustrated that an imbalance in Notch and Wnt/β-catenin signaling due to loss of the Notch-regulated ankyrin repeat protein (Nrarp; Q91ZA8) leads to premature vessel regression, likely as a consequence of reduced cell proliferation [9]. How physical forces and signaling pathways collectively stabilize or disrupt vessel connections remains unknown.

Here we investigate with high resolution the cellular mechanisms contributing to vessel regression in mouse and zebrafish. We find that vessel regression in mouse developmental angiogenesis is largely cell-death independent. We demonstrate that, rather, vessel regression involves dynamic rearrangement of endothelial cells, which migrate from regressing vessel segments to integrate in neighboring vessels. We propose that developmental vessel regression involves four discrete steps: (1) selection of the regressing branch, (2) lumen stenosis, (3) endothelial cell retraction, and (4) resolution of the regressing vessel segment. At the cellular level, we observe junctional arrangements similar to those found during vessel anastomosis, suggesting that vessel regression resembles morphologically anastomosis in reverse. Furthermore, we propose that endothelial cell nucleus-to-Golgi axial polarity predicts migration patterns at sites of vessel regression in vivo, and that differential flow/shear patterns in juxtaposed vessels drive asymmetries in cellular movements, thereby promoting stabilization of high-flow and regression of low-flow vessel segments.

## Results

### Endothelial Cell Death Is Not the Main Driver of Developmental Vessel Regression

**Fig 1:**
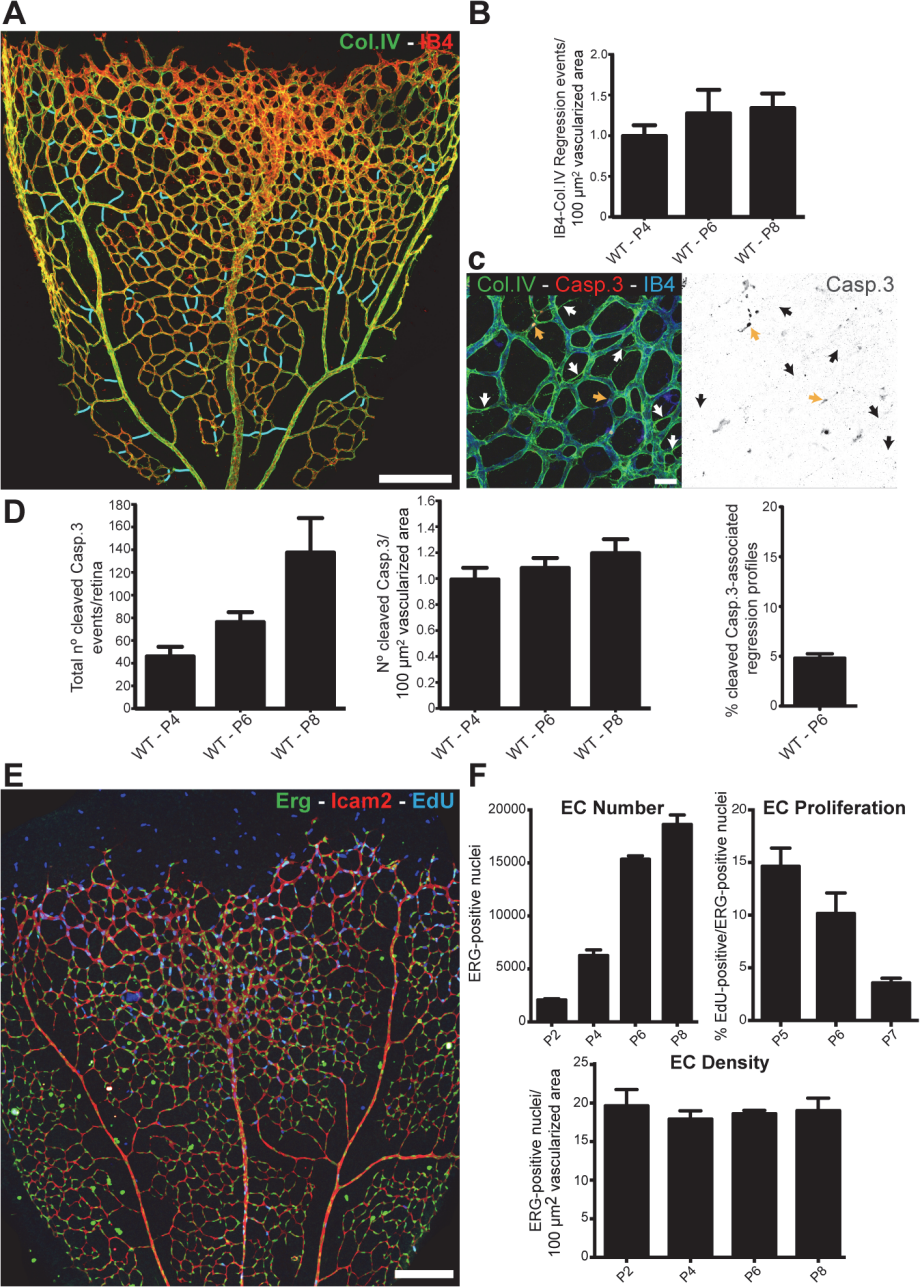
Developmental vessel regression does not depend on endothelial cell death. A, Overview of a wild-type postnatal day 6 (P6) mouse retina highlighting all regression profiles (blue lines). Regression profiles are vessel segments with collagen IV-positive vessel segments and negative for IsolectinB4. B, Quantification of number of regressing segments at P4, P6, and P8 retinas per vascularized area. C, Representative image of a P6 mouse retina labeled with Col.IV (green), cleaved caspase-3 (red) and IsolectinB4 (blue) showing regression profiles (white arrows) associated with cleaved caspase-3-positive cells (yellow arrows). D, Quantification of total numbers of cleaved caspase-3 events in entire P4, P6, and P8 mouse retinas, normalized for 100 μm^2^ of vascularized tissue. At P6, only 4.82% ± 0.76 (*n* = 5 retinas) of regression events are associated with caspase-3-positive labeled endothelial cells. Data given as mean ± SD. E, Confocal images of P6 wild-type retinas after 4h EdU-treatment (EdU, blue), endothelial cell nuclei (Erg, green) and blood vessels (ICAM2, red). F, Quantification of total number of endothelial cells, percentage of ETS related gene (Erg)- and 5-ethynyl-2'-deoxyuridine (EdU)-positive cells to total number of endothelial cells, and number of endothelial cells per vascularized area at specified mouse retina developmental stages. Mean ± SEM; *n* = 4 mice, 2 litters. Scale bars (A and E: 200 μm; C: 25 μm). The data used to make this figure can be found in S1 Data.

Remodeling of primitive vascular networks through substantial regression of vessel segments is detectable as empty type IV collagen (Col.IV) matrix sleeves (Figs 1A and S1). The number of regression points per vascularized area increased only slightly as remodeling progressed during postnatal stages (Fig 1B), suggesting that regression profiles have a limited lifetime and therefore do not accumulate. Over the period analyzed, vessel regression was proportional to the total area vascularized. Programmed, vessel regression is mediated by endothelial cell apoptosis and correlates with macrophage activity [3,10]. To analyze whether developmental vessel regression in the retina involves endothelial cell death, we quantified the number of apoptotic endothelial cells (cleaved caspase 3) at different postnatal stages. We observed a mean of 78.1 ± 7.0 events (±SD, *n* = 8) in a whole 6-d post-natal (P6) retina, comprising approximately 16,000 endothelial cells (Fig 1C–1F and S1 Data). Although the total numbers of apoptotic endothelial cell events per retina increased over time, the ratio of apoptotic endothelial cells and the numbers of endothelial cell per vascularized retinal area remained surprisingly constant (Fig 1D–1F and S1 Data). Moreover, at P6, only 4.82% ± 0.76 (mean ± SD, *n* = 5) of the abundant regression profiles were associated with active endothelial cell apoptosis (Fig 1D and S1 Data). Thus, 95% of the regression events were not directly associated with endothelial apoptotic events, suggesting that vessel regression initiation or completion is largely unrelated to apoptosis in physiological vascular development in the mouse retina.

### Developmental Vessel Regression Resembles Anastomosis in Reverse

**Fig 2:**
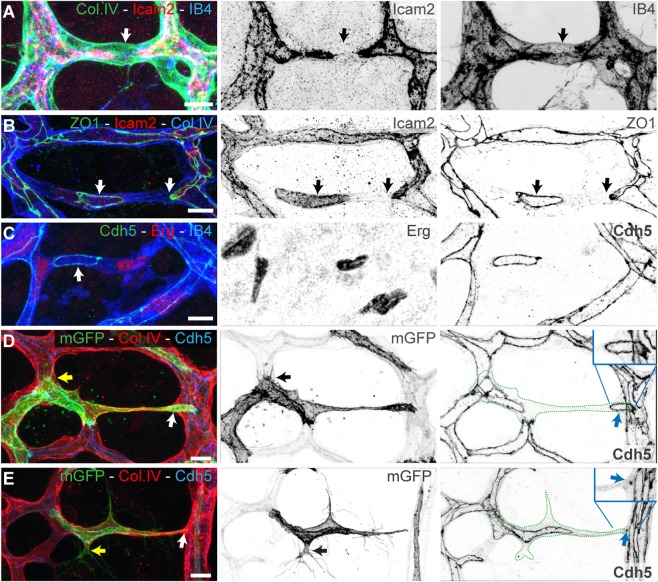
Developmental vessel regression resembles anastomosis in reverse. **A–C,** Immunostaining for lumen (ICAM2), junctions (ZO1 and Cdh5), blood vessels (IB4), and basement membrane (Col.IV) shows that lumen breakage (arrows in **A**) and junction disconnection (arrows in **B and C**) is an early step in vessel regression. **D and E,** Single-cell labeling using Cre-induced expression of membrane-bound GFP (mGFP) shows polarized morphology of activated endothelial cells with filopodia projections (yellow arrows in in **D** and **E**). Endothelial cells (green dotted-lines) bridge two or more vessel segments in the regressing vessel, showing rings or points of junctional connection (blue arrows in in **D** and **E**). Scale bars (**A–E**: 10 μm).

Analyzing endothelial cell configurations in regressing vessels by co-staining for intercellular adhesion molecule 2 (ICAM2) (P35330) (marking the apical/luminal endothelial cell membrane [11]), Col.IV, and isolectin B4 (IB4), we identified disrupted lumen as the first visible sign of vessel regression (Fig 2A). It was demonstrated in zebrafish that vessel regression in the brain vasculature was influenced by vessel perfusion [6]. Indeed, following perfusion of rhodamin-conjugated concanavilin-A in mouse pups, we observed that lumen disconnections were preferentially observed in rhodamin-negative vessel segments (S2A Fig). Co-labeling with vascular endothelial (VE)-cadherin (P55284) or zona occludens protein 1 (ZO1) (P39447) illustrated that the usual continuous junctions lining stable vessels as parallel lines are disrupted in branches with interrupted lumen. Instead, the junctions form isolated ring structures, often surrounding a patch of apical endothelial membrane without contact to the lumen in neighboring vessels (Fig 2B and 2C; S1 and S2 Movies). Such junctional arrangements surrounding apical membrane patches have been previously reported in early stages of lumen formation during anastomosis in the dorsolateral anastomotic vessel (DLAV) of zebrafish embryos [12]. At regression sites, they are additionally surrounded by a continuous Col.IV basement membrane (Fig 2B), suggesting that this configuration of lumen and cell junctions represents an intermediate step common to both anastomosis and regression. Indeed, by mosaic single-cell labeling using low-dose tamoxifen-induced Cre-mediated activation of membrane enhanced green fluorescent protein (eGFP) expression, we observed that endothelial cells in regressing branches extend numerous filopodia, similar to fusing endothelial tip cells, representative of activated endothelium (Fig 2D and 2E and S2 Movie). This suggests that endothelial cells could be actively migrating within the remodeling vascular plexus, and not only at the vascular sprouting front. In agreement, looking at the patterns of endothelial cell distribution in chimeric mouse retinas, we observed a lack of cohesive clonal expansion of proliferating endothelial cells (S2B Fig) [13]. These observations, although derived from static images in the retina, are nevertheless consistent with the idea that rearrangements of endothelial cells contribute to remodeling and possibly drive regression.

### Endothelial Cell Axial Polarity in Vessel Regression

**Fig 3:**
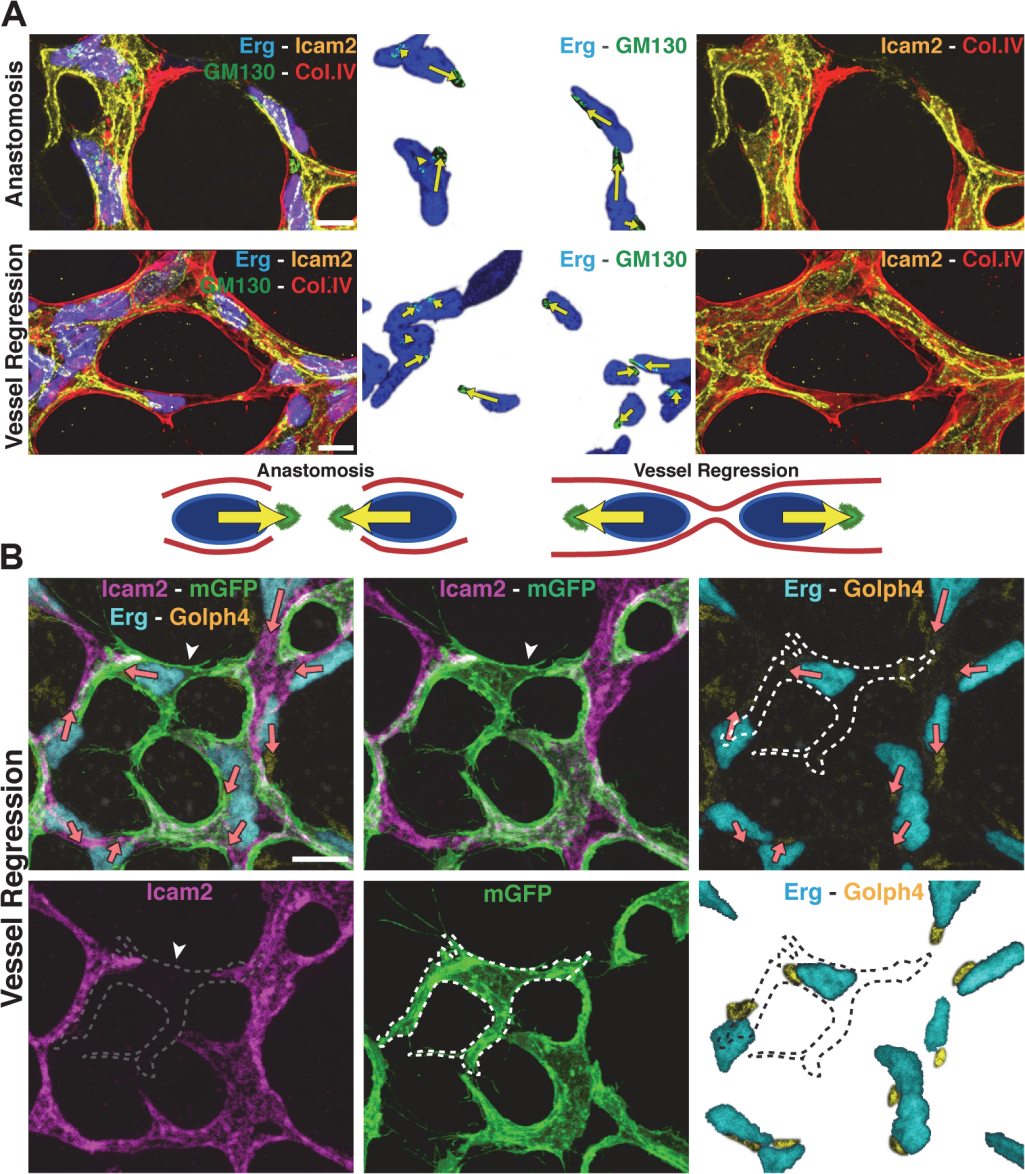
Disorganized endothelial cell polarity correlates with vessel regression. A and B, Polarization of endothelial cells in a wild type (WT) P6 retina vascular network labeled for endothelial cell nuclei (Erg), basement membrane (Col.IV) lumen (intercellular adhesion molecule 2 [ICAM2]), and Golgi (130 kDa cis-Golgi matrix protein [GM130] or Golgi integral membrane protein 4 [Golph4]). Distance from the center of mass of each endothelial nucleus to the corresponding Golgi is used to draw a yellow (A) or pink (B) arrow, indicative of front–rear (axial) polarity. A, In anastomosis, endothelial polarities point towards each other. In regression, endothelial cells polarities point towards the neighboring vessel segments. Images were segmented for visualization purposes; original images can be found in S4 Fig B, Representative image of stochastic cell labeling using inducible Cre-lox mediated expression of membrane-bound GFP (mGFP), revealing the morphology of single endothelial cells in regressing vessels (white arrowhead) in combination with the axial polarity assessment (pink arrows). The white dotted line outlines one cell in a regression profile (lacking ICAM2), which is in contact with multiple vessel segments and shows an activated morphology with numerous filopodia. Scale bars (A and B: 10 μm).

To gain a better understanding of directionality and coordination of cell movements, we investigated endothelial cell polarity along the axis of vessel segments in the remodeling plexus. In vitro, endothelial cells position their Golgi apparatus ahead of the nucleus in the direction of migration [14]. Using the endothelial-specific transcription factor Erg to label endothelial nuclei, the Golgi marker Golgi integral membrane protein 4 (Golph4) (Q8BXA1), together with lumen and Col.IV labeling, we determined endothelial cell nuclear shape and Golgi location in the mouse retina at P6 (Figs 3A and S3A). We established the distance between the center of mass of the endothelial nuclei and the position of the corresponding Golgi apparatus as a vector representing axial cell polarity (Fig 3A). Live imaging in transgenic zebrafish embryos confirmed the dynamic correlation between axial Golgi polarization and directional endothelial cell migration (S3 Movie). The Golgi apparatus of two anastomosing tip cells generally pointed towards the point of contact (Fig 3A), while in regressing vessels the Golgi position arrangements were reversed, suggesting that cells migrate away from each other during regression (Fig 3A and 3B). We therefore propose that regression in developing vascular plexuses is a cell migration–driven process, resembling the cellular events occurring during anastomosis in reverse order.

### A Four-Step Model for Blood Vessel Regression

**Fig 4:**
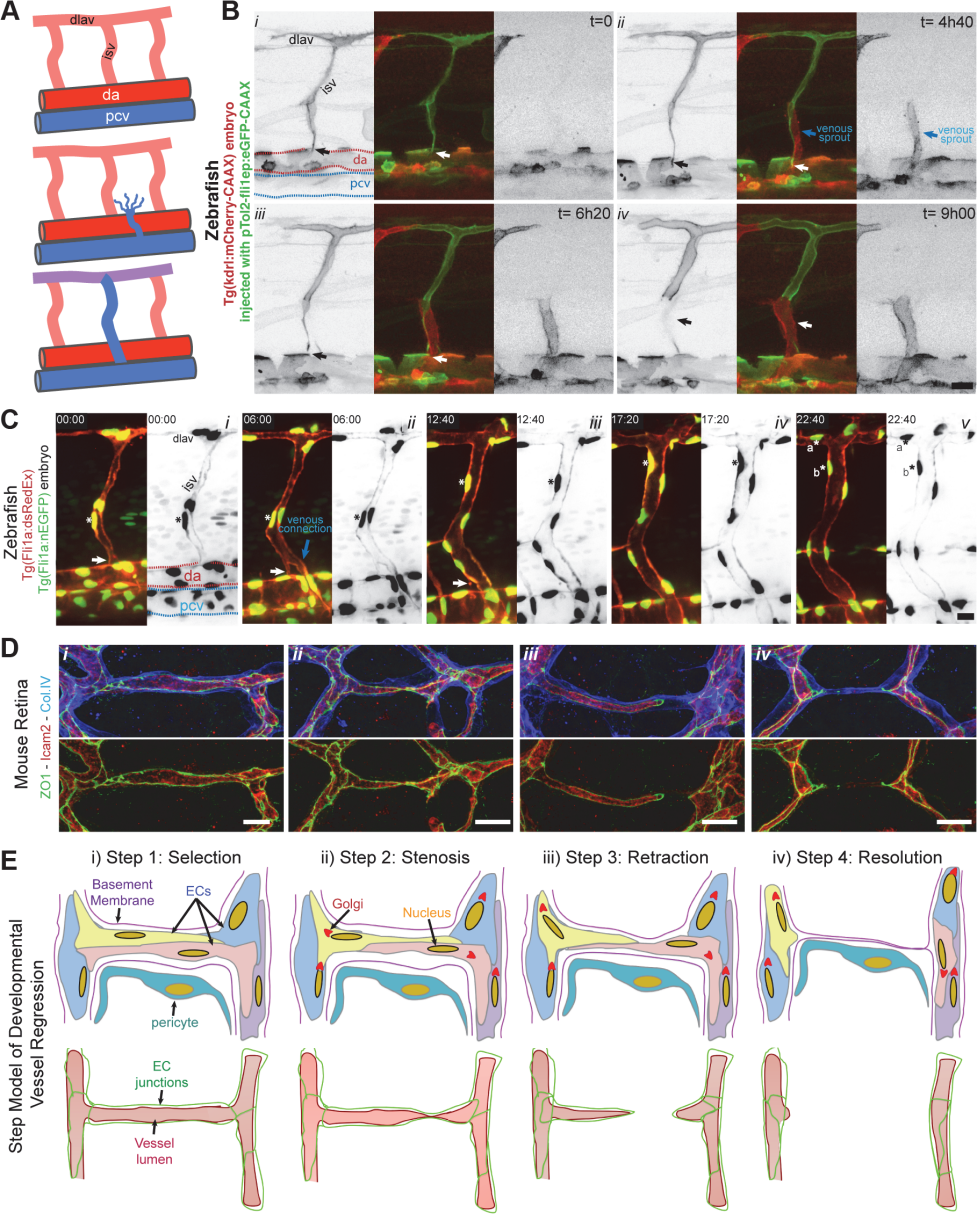
Extensive cell rearrangements drive developmental vessel regression. **A,** Schematic of ISV disconnection from the aorta. **B,** S4 Movie still images from time-lapse confocal imaging at 48 h post-fertilization of a transgenic Tg(*kdrl*:mCherry-CAAX) zebrafish embryo injected with pTol2:fli1ep:eGFP-CAAX, showing the dynamic behavior of endothelial cells during the process of intersegmental vessel regression (white arrow), triggered by the anastomosis of a venous sprout (blue arrow). **C,** S5 Movie still images from time-lapse confocal imaging at 48 h post-fertilization of a transgenic Tg(*Fli1a*:*dsRedEx*); Tg(Fli1a:nEGFP) zebrafish embryo showing the dynamic behavior of endothelial cell nuclei during vessel regression (white arrow). The regressing endothelial cell (asterisk) is viable and undergoes mitosis a later stage, originating two daughter endothelial cells (asterisk a and b). **D,** Confocal images of regression profiles in a wild-type P6 mouse retina labeled with lumen (ICAM2), junctions (ZO1), and basement membrane (Col.IV). Vessel segments range from a normal stable vessel segment (left panel), stenosis lumen/junction, disconnected lumen, and complete absence of lumen (right panel). **E,** Proposed four-step model for vessel regression. Step 1: selection of the regressing branch, Step 2: lumen stenosis in the regressing vessel, Step 3: junction/lumen remodeling during endothelial cell retraction, and Step 4: integration of regressing endothelial cells in neighboring vessel segments leaving an empty basement membrane. (dlav: dorsolateral anastomotic vessel; isv: intersegmental vessel; da: dorsal aorta; pcv: posterior cardinal vein). Scale bars (**B and C**: 20 μm; **D**: 10 μm).

To directly observe cell dynamics during the process of vessel regression, we studied regression of intersegmental vessels (ISVs) during remodeling of arterial to venous ISVs in the zebrafish embryo. After the first angiogenic phase, ISVs are originated from endothelial sprouts arising from the aorta. A second angiogenic phase occurs at a later stage in development, in which secondary sprouts arising from the posterior cardinal vein (PCV) either connect with the arterial ISVs, triggering disconnection from the aorta, or instead form precursors of the zebrafish lymphatic system (Fig 4A) [15]. We generated mosaic endothelial expression of membrane-bound eGFP in Tg(*kdrl*:*mCherry-CAAX*) embryos to observe the dynamics of single endothelial cells during regression of the connection of ISVs to the aorta (Fig 4B and S4 Movie). Where venous sprouts connected to the ISV (Fig 4B *ii*), we observed subsequent disconnection and retraction of the arterial cells from the aorta (Fig 4B *iii*, *iv*). Similar to the mouse retina observations, the disconnection of the ISVs occurred without evident endothelial cell death (S4 Movie). Cell tracking using a zebrafish transgenic line labeling endothelial nuclei (Fig 4C and S5 Movie) also revealed migration of cells with no sign of apoptosis during and after regression. In the shown example, the regressing cell proliferates after regression (Fig 4C *v*), confirming that cells involved in regression remain active and viable. When comparing vessel regression in the mouse retina and the zebrafish ISV, we could observe striking similarities in the cellular arrangements during the different phases of vessel regression (Fig 4D). On the basis of these observations, we schematized the cellular and junctional rearrangements underlying vessel branch regression (Fig 4E). We propose that vessel regression entails four distinct steps: (1) an initial selection step, which precedes and triggers the morphological alterations during regression; (2) a stenosis step, in which the lumen is focally constricted or collapsed; (3) a retraction step, in which endothelial cells migrate and retract processes, associated with junctional remodeling; (4) a resolution step, which comprises the final loss of any endothelial processes in this branch, leaving only basement membrane and pericyte(s) behind (Figs 4E and S4).

### Vessel Regression Correlates with Lower Endothelial Cell Axial Polarities in Low-Flow Vessel Segments

**Fig 5:**
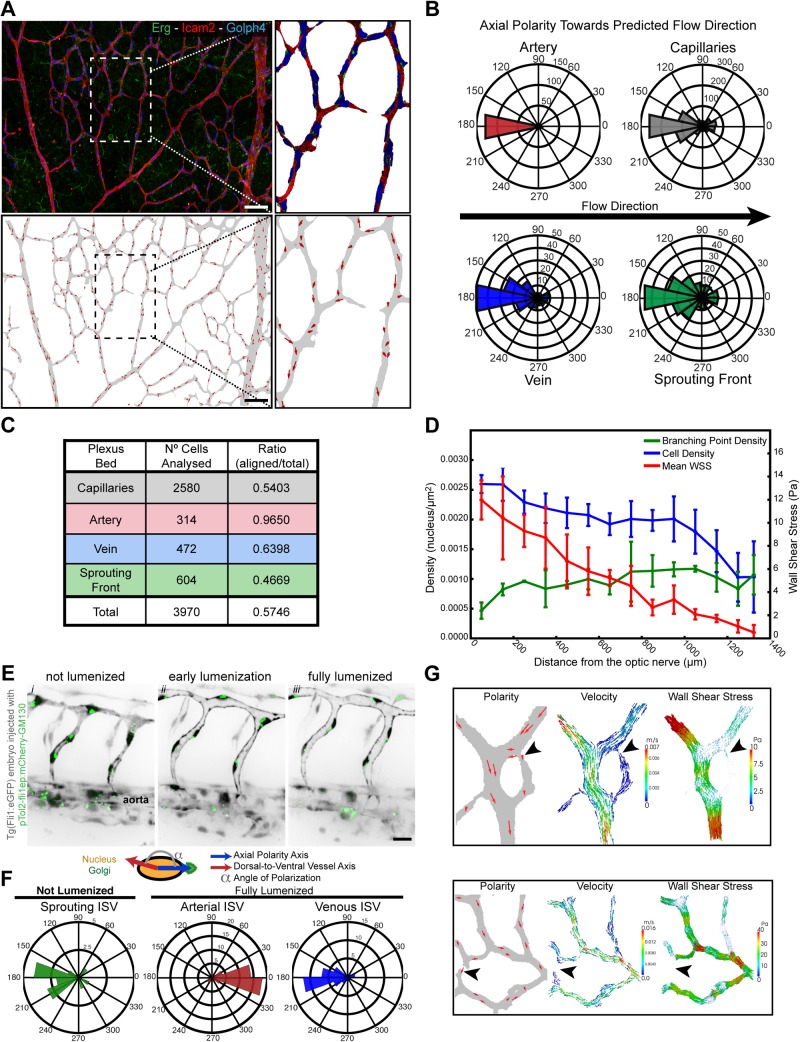
Coordinated polarity induced by high flow triggers vessel regression. **A,** Overview of the axial polarization pattern of endothelial cells in a WT P6 retina vascular network labeled for endothelial cell nuclei (Erg), lumen (ICAM2), and Golgi (Golph4), and corresponding image segmentation of the vascular plexus in (a), highlighting the lumen of blood vessels (grey), and the axial polarity of all endothelial cells (red arrows). **B,** Analysis of the endothelial axial polarity angle in the main vessels, correlated to predicted blood flow direction by the rheology in silico model. Endothelial cells robustly position their Golgi apparatus against the blood flow in all vascular regions analyzed. **C,** Quantitative analysis of the percentage of endothelial cells polarized at 180°(±45°) compared to the flow direction in the different vascular beds (*n* = 3 retinas). **D,** Quantitative analysis of cell density, mean wall shear stress and branching point density in P6 mouse retina vascular plexus (*n* = 3 retinas). **E,** S6 Movie still images from time-lapse live imaging of a Tg(fli1a:eGFP) zebrafish embryo (grey) injected with pTol2:fli1ep:mCherry-GM130 (green). **F,** Quantification of endothelial cell axial polarity in ISVs showing dorsal axial polarization during the sprouting phase (not lumenized). In stable ISVs, endothelial shows significantly enriched dorsal or ventral axial polarity when in venous or arterial ISVs, respectively, corresponding to polarization against the predicted blood flow direction. **G,** Representative images of axial polarity and color-coded representation of the rheology prediction for velocity and wall shear stress in the corresponding vessel segments. Axial polarity length correlates with higher levels of luminal shear stress. In low shear vessels endothelial cells show decreased polarization and tend to point towards high flow vessel segments (black arrows). Scale bars (**A**: 50 μm; **E**: 20 μm). The data used to make this figure can be found in S1 Data.

Stimulated by our analysis of the axial endothelial polarity in vessel regression and the importance of haemodynamics in regulating blood vessel pruning in the zebrafish brain [6], we took advantage of the newly developed approach for the computation of haemodynamic forces in mouse retinal vascular networks [16] to investigate the correlation between endothelial axial polarity and blood flow patterns in the mouse retina (Figs 5A and S5). Interestingly, in the retinal plexus, axial polarity vectors were largest in high flow vessels, such as arteries and arterioles, with very little variance and vectors pointing exclusively against the direction of blood flow (Figs 5B, 5C, and S6). Also in veins, polarity was generally directed against the flow, but the vectors were, in general, smaller (Figs 5B, 5C, and S6). Surprisingly, even closer to the retinal sprouting front and distant from the feeding arteries, where flow and shear levels are predicted to be low through our simulations, endothelial axial polarity is still significantly directed against flow (Fig 5A–5C, S1 Data, and S6 Fig). Linear regression analysis identified a strong correlation between increasing wall shear stress and polarization (analyzed as scalar product of polarity and shear vectors, S6 Fig and S1 Data). In order to better understand the relationship between flow-induced shear and vascular parameters across the retina, we performed unbiased combinatorial quantitative analysis of wall shear stress, cell density, and branchpoint density as a function of the distance from the optic nerve (Fig 5D, S1 Data). With increasing distance from the optic nerve, branchpoint density increased, reflecting the transition from the more remodeled to the unremodeled plexus. In parallel, the wall shear stress levels decreased with distance from the optic nerve. However, surprisingly, endothelial cell density, measured as nuclei/μm^2^, was highest closest to the optic nerve and decreased towards the periphery. Thus, although the increase in branch points signifies a more ramified vascular plexus in the developing periphery, endothelial cell density is lower in this region. Conversely, the highest wall shear stress found in the most central and remodeled area correlated with higher cell density (Fig 5D, S1 Data), indicating that the transition from primitive to remodeled plexus is not driven by cell loss. Given that minimal proliferation is detected in these more central areas (Fig 1E), the increased density of endothelial cells closer to the optic nerve independently argues against endothelial cell apoptosis as a driver of vascular remodeling. Instead it would be consistent with cells incorporating into higher flow segments as low-flow segments regress. To directly observe endothelial axial Golgi-to-nucleus polarity under the influence of blood flow, we analyzed transgenic zebrafish embryos expressing transiently mCherry-GM130, a Golgi-specific protein, during the process of lumenization and blood flow onset in intersegmental vessels. Endothelial cells in ISVs without continuous lumen showed variable axial polarities, with cells positioning their Golgi apparatus in the direction of migration, occasionally directed towards the aorta, however, mostly directed towards the DLAV (Fig 5E and 5F and S6 Movie). Interestingly, as ISVs formed a continuous lumen and flow is established, endothelial cells redirected their axial polarity towards the aorta and against the blood flow direction. Thus endothelial cells respond dynamically to the onset of flow and rapidly redirect their Golgi against the direction of flow (Fig 5E and S6 Movie). Quantification of axial polarization in ISVs demonstrates that during the sprouting phase arterially connected endothelial cells show dorsal polarization, which is reversed in mature arterial ISVs, to point against the flow direction (Fig 5F, S1 Data). In stable venous ISVs, axial polarity points dorsally, i.e., against the flow direction (Fig 5F, S1 Data).

Interestingly, looking at regions in the retinal vascular network showing coordinated endothelial axial polarities—around arteries and first order branches—with clear differences in wall shear stress levels between adjacent vessel segments, we observed a strong correlation amongst the lower wall shear stress vessels segments and the presence of endothelial cells with very low axial or misaligned polarity vectors (Fig 5G; S5 and S6 Movies).

## Discussion

### Cellular Principles of Vessel Regression

**Fig 6:**
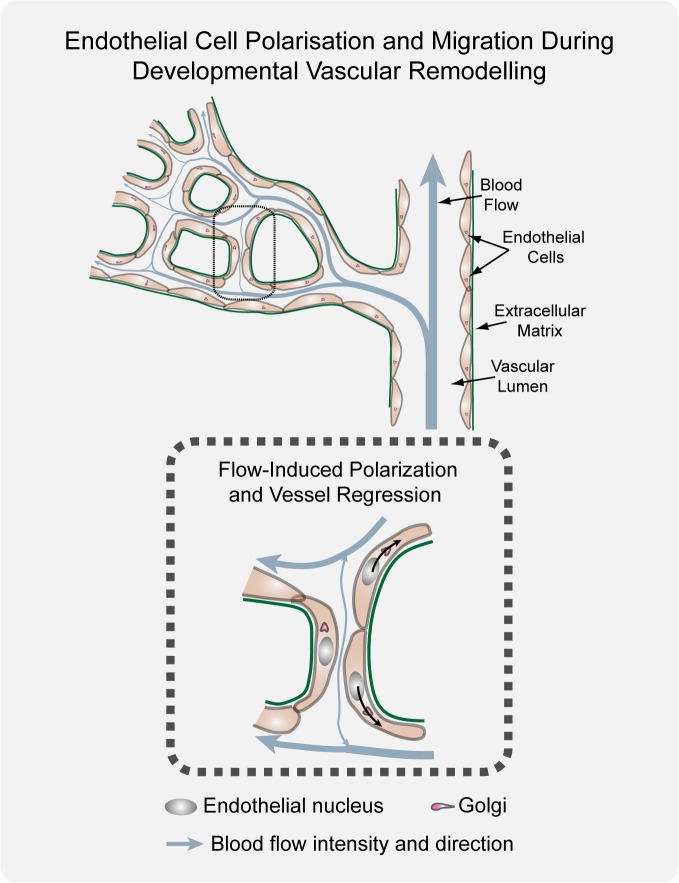
Working model for flow-induced remodeling through directional migration. Schematized prototypic vessel network in a developing retina. Endothelial cell axial polarity is indicated by Golgi position; flow direction (arrows) and velocity (thickness), producing luminal membrane shear stress, are depicted by light blue lines. Vessels in the distal primitive plexus are exposed to low, oscillatory, or no-flow, and vessels closer to developing arteries are exposed to higher blood flow velocities. High blood flow leads to increased levels of shear stress, which induces robust polarization of endothelial cells against flow. Increasing flow asymmetries between juxtaposed vessel segments trigger endothelial migration away from low flow regions (black arrows), inducing vessel segment regression.

Based on the present observations, we propose that endothelial cells migrate and rearrange dynamically, not only in sprouts, as shown previously [13,17,18], but also in the newly formed and the remodeling plexus. In the primitive plexus, this migratory behavior lacks an overt directionality and is thus balanced, enabling a symmetric distribution of cells throughout all segments, thus forming a uniform primitive plexus. When flow creates sufficient high shear forces on the endothelial luminal surface, this new directional force breaks the symmetry and drives polarization against the blood flow direction. This polarization directs migration of cells in low-flow or oscillatory flow segments towards the high flow segments, thus destabilizing the segment. As a consequence, there will be a net movement of cells out of the low-flow branch into the higher flow branch, thus leading to regression of the former and stabilization of the latter. Similarly, live-imaging in zebrafish brain vasculature demonstrated that regressing vessel segments exhibit low flow, which decreased irreversibly prior to the onset of regression [6]. Interestingly, the set value for shear stress when vessels enter the regression program is variable, but seems to depend on shear stress levels on juxtaposed vessel segments [6]. Taken together, we propose that increasing flow asymmetry between juxtaposed vessel segments is the trigger for developmental vessel regression (Fig 6). At the regressing segment, low-flow conditions are insufficient to establish strong continuous cell polarity within the segment. Where these cells connect to and sense higher flow in neighboring segments, the resulting polarity will lead to “attraction” of cells into the high-flow segment. In principle, axial polarity can be a component of directional cell migration or a consequence of the shear forces exerted onto the cell. Thus the migratory polarization and flow-induced polarization may be distinct events. Recent work by the Siekmann team, however, identified movement of endothelial cells in a remodeling plexus from the vein to the artery [19], thus against the predicted direction of flow. Therefore, it is also possible that the migration and flow-induced polarity events are tightly linked. The observed vessel stenosis could also be a trigger of poor perfusion, and thus polarity, or the consequence of the migratory behavior and attraction of the cells out of this segment and into the neighboring one.

Intriguingly, we noted an association of apoptotic events with long regressing vessel segments, especially when disconnecting from retinal arteries. We hypothesize that apoptosis during developmental vessel regression might be associated with a failure of endothelial cells to integrate into neighboring vessel segments.

The observed lumen stenosis may result from active endothelial contraction [8], RhoA over-activation [20], or passive lumen collapse, and conceivably could also be triggered by endothelial cell retraction. In contrast, the programmed regression of fetal ocular vessels is triggered by induced single endothelial cell apoptosis, leading to flow stasis, followed by synchronous endothelial cell apoptosis [3,4]. Similarly, experimental oxygen-induced vessel regression involves widespread endothelial cell apoptosis in the retinal vasculature [21]. Therefore, two distinct mechanisms for initiation and completion seem to be operating during vessel regression depending on the context, extent and biological requirement; (1) endothelial cell apoptosis for programmed regression of entire networks, and (2) endothelial cell migration for angiogenic remodeling. Defining the molecular mechanisms regulating each step will be critical to fully understand the process of vessel regression.

### Blood Flow Acts As a Coordinator of Endothelial Cell Polarization

The profound motility and rearrangement of endothelial cells in the immature vascular plexus [6,13,22] implies that endothelial cells need to coordinate their cellular movements in order to maintain vessel integrity and vessel connections. In a wide range of developing tissues, the orientation and coordination of cells is dependent on intrinsic and extrinsic factors, such as morphogen gradients, extracellular matrix adhesion, cell junctions, physical forces, and cell-to-cell communication, which all participate in the correct cell polarization and coordinated cell migration [23,24]. Here, we show that endothelial cell Golgi polarity predicts migration patterns at sites of vessel regression in vivo. In the primitive plexus, where flow is low, endothelial axial polarity is less apparent, suggesting that cell movement is less directional or less collectively aligned. We hypothesize that this movement of cells maintains the primitive plexus in a metastable state of symmetry, with cells evenly distributed throughout all vessel segments.

### Emerging Concepts in Developmental Vascular Remodeling

The emerging conceptualization of vessel regression favors a model in which endothelial cells proliferate to provide sufficient numbers to support formation of the primitive plexus and are then rearranged and re-used in the process of making a functional vascular plexus to meet regional demands. Several observations support this model: (1) low number of apoptotic endothelial cells associated with regressing vessel segments; (2) a surprisingly similar number of endothelial cells per vascularized area before and after remodeling, yet with highest cell densities in central and more remodeled areas; (3) cells actively migrate from regressing vessel segments and integrate in the juxtaposed vessel network; (4) decreased proliferation of endothelial cells lead to excessive vessel regression, with cells stretching over long distances [9]. In the chick yolk sac, vessels disconnecting from the vitelline arteries are re-used for establishing new vessel connections with neighboring veins [25], and also in the mouse yolk sac, endothelial cells move from smaller into larger caliber vessels, contributing to remodeling [26].

What drives the rearrangements of cells in the primitive plexus and how flow in one segment initiates regression in another is poorly understood. Recent results show that differential VE-cadherin dynamics drive cell rearrangements in the vascular sprout [18]. Cells with higher vascular endothelial growth factor (VEGF) signaling and lower Notch activity show increased mobility by displaying a larger mobile fraction of VE-cadherin at their junctions. Whether this also holds true for events during regression is unclear. However, given that Notch is also active in remodeling [8,27], VE-cadherin is a component of endothelial cell-to-cell and fluid shear stress force sensing [28], and that VE-cadherin is implicated in coordinating endothelial polarity in collective migration [14], it is tempting to speculate that rearrangements in the primitive plexus involve Notch signaling as a driver of local differences.

## Materials and Methods

### Ethics Statement

All studies and procedures involving animals were in strict accordance with the European and United Kingdom Regulation for the Protection of Vertebrate Animals used for Experimental and other Scientific Purposes Animal procedures in accordance with the Home Office Animal Act 1986 under the authority of the Project License PPL 80/2391. Suffering of the animals was kept to a minimum; no procedures inflicting pain were performed.

### Mice and Treatments

For perfusion fix experiments, P6 pups were anaesthetized via IP injection of 0.1 ml/10 g of Ketaset/Hypnovel mix. Mouse pups were then perfused, via left ventricle intracardiac puncture, with room temperature PBSa, followed by 1% PFA solution, and finally perfused with Rhodamine labeled Concanavalin A (Vector Labs) in PBSa (0.05 mg/ml) at room temperature for 10 min. Eyes were thereafter collected for further analysis. For chimeric retina experiments, at 3.5 d post-coitus (dpc), embryos from PDGFb-iCreER; Rosa26mTmG mice were used to isolate ES cells, which were cultured in standard ES cells media with the MEK inhibitor PD0325901 (Stemgenet), as described previously [29]. ES cells were characterized and injected into 3.5-dpc staged Balb/cOlaHsd wild-type embryos and re-implanted into pseudopregnant foster females using standard protocols [30]. Newborn pups were injected intraperitoneally with Tamoxifen (Sigma; 20 μl/g of 1 mg/mL solution) at P3 before eyes were collected at P6. For endothelial proliferation assessment in the retina, mouse pups were injected IP 4 h before collection of eyes with 20 μl/g of EdU solution (0.5 mg/mL; Invitrogen, C10340).

Oxygen-dependent vessel obliteration was achieved using two different regimes of hyperoxia. At P4 (regime 1) or P7 (regime 2) pups were place in 70% oxygen chamber until P6 (regime 1) or P12 (regime 2). Animals were sacrificed immediately after hyperoxia treatment and processed for retinal vasculature analysis.

Animal procedures were performed in accordance with the Home Office Animal Act 1986 under the authority of project license PPL 80/2391.

### Zebrafish Experiments and Time-Lapse Imaging

The following transgenic zebrafish strains were used: Tg(fli1a:eGFP) [31]; Tg(fli1a:nEGFP)^y7^ [32]; and Tg(Fli1a:dsRedEx)^um13^ [33]. Transgenic zebrafish embryos Tg(fli1a:eGFP)^y7^ were injected with 45 ng/μl of plasmid DNA pT2Fliep-mCherryGM130 at one-cell stage. Embryos were raised at 28°C and screened for transient expression at ~30 hpf. Positive embryos were anaesthetized in 1x tricaine (0.08%) and mounted in a 35 mm glass bottom petri dish (0.17 mm, MatTek), using 0.7% low melting agarose (Sigma) containing 0.08% tricaine and 0,003% PTU. Time-lapse analysis was performed using a Leica TCS SP5, an Andor Revolution 500 spinning disk or a Zeiss LSM710 confocal microscope, with 20x or 40x objectives.

### Immunofluorescence

Primary and secondary antibodies are listed in S1 Table. At desired stage of development, mouse eyes were collected and fixed 5 h in ice-cold 2% paraformaldehyde (PFA) in PBS for 5 h at 4°C. Thereafter retinas were dissected in PBS. Blocking/permeabilisation was performed using Claudio’s Blocking Buffer (CBB), consisting of 1% FBS (Gibco), 3% BSA (Sigma), 0.5% triton X100 (Sigma), 0.01% Na deoxycholate (Sigma), 0,02% Na Azide (Sigma) in PBS pH = 7.4 for 2–4 h at 4°C on a rocking platform. Primary and secondary antibodies were incubated at the desired concentration in 1:1 CBB:PBS at 4°C overnight in a rocking platform. When using two rabbit primary antibodies, such for Erg and Golph4 immunofluorescence images in Figs 3, 5, S4, and S5, after first incubation with Erg primary and corresponding secondary, an additional step of incubation with donkey anti-rabbit Fab fragments (1:100, Jackson’s Laboratories) followed by 15 min fixation with 4% PFA was performed prior the incubation with Golph4 primary antibodies, in order to avoid intensive cross-reaction between the two primary antibodies. DAPI (Sigma) was used for nuclei labeling. Retinas were mounted on slides using Vectashield mounting medium (Vector Labs, H-1000). For imaging we used a Carl Zeiss LSM780 scanning confocal microscope.

### Quantification Measurements and Statistical Analysis

Complete high-resolution three-dimensional (3-D) rendering of whole mount retinas were acquired using a LSM780 laser-scanning microscope (Zeiss). Tiled scans of whole retinas were analyzed with Imaris (Bitplane) or ImageJ. Proliferation of endothelial cells was measure by quantifying the total number of endothelial cell nuclei (labeled by Erg immunostaining) positive for EdU staining in 3–5 20x objective images, in regions containing the sprouting front, and dividing by the total area of vascularized tissue. Quantification of apoptosis in regression profiles was measured as the number of regression profiles positive for cleaved caspase-3 and divided by the total number of regression profiles in regions used for quantification, and given as percentage.

### Mouse Retina Rheology Model

Details of the computational rheology model used to study flow patterns in the developing mouse retina can be found in [34]. Briefly, retinal vascular plexuses were stained for ICAM2 and imaged following the above-mentioned protocol. The resulting images were post-processed with Photoshop CS5 (Adobe) in order to isolate the luminal region of interest, which was further processed with MATLAB (The MathWorks, Inc.) in order to extract the image skeleton and compute vessel radii along the network. Based on the computed image skeleton and radii, a three-dimensional triangulation of the plexus luminal surface was generated with VMTK (Orobix srl). The computational fluid dynamics software package HemeLB (see [17] for more details) was used to compute high-resolution estimates of pressure, velocity, and shear stress across the domain. Flow visualization was generated with Paraview (Kitware, Inc.), and post-processing of the results was performed with custom-made Python scripts [34].

## Supporting Information

S1 Data This file contains data for Figs 1C, 1D, 1F, 5B–5D, 5F, and S6. (XLSX)

S1 Fig Vessel remodeling involves a continuous process of vessel regression. **A,** Overview of wild-type P6 and P13 mouse retinas highlighting all regression profiles (blue lines). Regression profiles are vessel segments with Col.IV-positive vessel segments and negative for ICAM2 staining or presenting a breakage in the continuity of the luminal staining. **B,** Typical basement membrane (Col.IV)-empty sleeve representing a regressed vessel segment (arrow) in a P6 retina. The basement membrane remains, while no lumenized vessel (ICAM2) or endothelial cell (IB4) is present. Scale bars (**A**: 200 μm; **B**: 20 μm).
(TIF)

S2 Fig Developmental vessel regression involves dynamic cell rearrangements and correlates with low/no-flow vessel segments. **A,** Confocal images of P6 wild-type retinas after fix-perfusion with rhodamin-conjugated concanavalin A (red). Retinas were stained for extracellular matrix (Col.IV) and the blood vessel lumen marker (ICAM2) and showed that regressing vessels correlated with rhodamin-negative vessel segments (arrows). **B,** Confocal images of chimeric retinas derived from injection of PDGFb-iCreER; R26mTmG ES cells into wild-type host blastocyst. Following tamoxifen-induced recombination at P2, retinas were collected at P6 and stained for endothelial nuclei (Erg) and a blood vessel lumen marker (ICAM2). Recombined endothelial cells (mGFP) were scattered throughout the vasculature and no-clonal expansion of endothelial cells in blood vessels could be seen. Scale bars (**a**: 50 μm; **b**: 200 μm).
(TIF)

S3 Fig Vessel regression resembles vessel anastomosis in reverse. **A,** Non-treated image shown in Fig 3A, showing all the channels in separate panels. Labels for stainings are shown in figure.
(TIF)

S4 Fig Cell rearrangements are the main driver of developmental vessel regression. **A,** Confocal images of several vessel segments, stained markers for endothelial cells (IB4), junctions (Cdh5), and endothelial cell nuclei (Erg) in a wild-type P6 mouse retina. Vessel segments are categorized according to configurations described in Fig 4E. **B,** Single-endothelial cell labeling, using Cre-induced expression of membrane-bound GFP (mGFP), shows polarized morphology of endothelial cells in different stages of vessel regression, as defined by the color-coded arrows. Scale bars (**A** and **B**: 10 μm).
(TIF)

S5 Fig Endothelial polarization patterns in the remodeling mouse retina. A, Wild-type P6 mouse retina stained for extracellular matrix (Col.IV), endothelial cell nuclei (Erg), blood vessel lumen (ICAM2) and Golgi apparatus (Golph4). B, Image segmentation of the vascular plexus of the mouse retina in (A), highlighting the lumen of blood vessels (grey), the regression profiles (green lines), and the nucleus-to-golgi (axial) polarity of all endothelial cells (red arrows). C, Color-coded shear stress map of in mouse retina vascular network in (A), predicted using a computational approach. D, Spatial representation of individual cells for each of the selected groups (artery, vein, capillary, and sprouting front) used for quantifications of axial polarity in Fig 5. Scale bars (A and B: 200 μm).
(TIF)

S6 Fig Distribution of scalar product of axial polarity and angle polarization related to wall shear stress levels. A, Graphs showing distribution of scalar products in function to wall shear stress levels for each vascular plexus. Scalar product corresponds to the product between length of the axial polarity vector and the cosine of the angle between the axial polarity vector and the flow direction vector. B, linear regression analysis of positive (polarized with flow) and negative (polarized against the flow) scalar product points for each endothelial cell nuclei. Gradient, R-value, and number of cells analyzed for each vascular bed is shown. *n* = 3 retinas. The data used to make this figure can be found in S1 Data.
(TIF)

S1 Movie 3-D view of vessel regression. 3-D rotation visualization confocal microscopy stack image of endothelial cells in the remodeling vascular plexus. Endothelial cell nuclei are labeled with Erg (red), endothelial junctions with VE-cadherin (green), and all nuclei with DAPI (grey). Video highlights a vessel regression, at the center of the field of view, where an endothelial cell is at one side of a regression profile with a ring-like structure of adherens junctions at the other end of the regression profile.
(MOV)

S2 Movie 3-D view of single endothelial cells in vessel regression. 3-D rotation visualization confocal microscopy stack image of endothelial cells in the remodeling vascular plexus. Single-cell labeling using Cre-induced expression of membrane-bound GFP (mGFP, green) shows endothelial cell shape and position in a regressing vessel segment, positive for collagen IV (red). The single endothelial cell at the center of the field of view connects two vessel segments and participates in the formation of two ring-like structures of adherens junctions (VE-cadherin, grey), illustrating the active cell rearrangements occurring during vessel regression. All nuclei are labeled with DAPI (blue).
(MOV)

S3 Movie Golgi positioning in a tip cell of an intersegmental vessel sprout. Time-lapse imaging of an intersegmental vessel sprout of a Tg(Fli:nuGFP)^y7^ embryo highlighting the endothelial cell nuclei (eGFP, green) injected with pT2Fliep-mCherry-GM130 plasmid, leading to chimeric labeling of the Golgi apparatus (mCherry, red). In a migrating tip cell, the Golgi apparatus localizes ahead of the nucleus, pointing towards the migration direction.
(MOV)

S4 Movie Dynamic endothelial cell rearrangements in intersegmental vessel regression. Time-lapse live imaging of transgenic Tg(*kdrl*:mCherry-CAAX) zebrafish embryo injected with pTol2:fli1ep:eGFP-CAAX. Two endothelial cells in the intersegmental vessel are attached to the aorta vessel. A venous sprout contacts an arterial intersegmental vessel and triggers regression of the previous connection to the aorta through dynamic rearrangements of endothelial cells, and without signs of endothelial apoptosis.
(MOV)

S5 Movie Dynamic observation of endothelial cell nuclei in intersegmental vessel regression. Time-lapse live imaging of a 48 h post-fertilization transgenic Tg(*Fli1a*:*dsRedEx*); Tg(Fli1a:nEGFP) zebrafish embryo showing the dynamic behavior of endothelial cell nuclei during vessel regression. The regressing endothelial cell is viable for several hours after disconnection from the aorta and undergoes mitosis at a later stage originating two daughter endothelial cells.
(MOV)

S6 Movie Axial polarity of endothelial cells correlates with increased flow. Time-lapse live imaging of a Tg(fli1a:eGFP) zebrafish embryo (red) injected with pTol2:fli1ep:mCherry-GM130 (green). Endothelial cells in the intersegmental vessels presents random axial polarity configurations prior to lumenization. As lumen is formed and flow coming from the aorta is established, endothelial cells start reorganizing their polarity axis towards the aorta, i.e., against the direction of blood flow. Time interval between frames is 10 min.
(MOV)

S1 Table List of antibodies used in the immunofluorescence studies. (DOCX)

## Abbreviations

Col.IV: collagen type IV
DLAV: dorsolateral anastomotic vessel
Dll4: delta-like ligand 4
eGFP: enhanced green fluorescent protein
EdU: 5-ethynyl-2'-deoxyuridine
Erg: ETS related gene
GM130: 130 kDa cis-Golgi matrix protein
Golph4: Golgi integral membrane protein 4
IB4: isolectin B4
ICAM2: intercellular adhesion molecule 2
ISV: intersegmental vessel
mGFP: membrane-bound GFP
Nrarp: Notch regulated ankyrin repeat protein
P: postnatal time
PCV: posterior cardinal vein
SD: standard deviation
VE-cadherin: vascular endothelial cadherin
VEGF: vascular endothelial growth factor
WT: wild type
ZO1: Zona occludens protein 1
