# Correction: Dynamic Endothelial Cell Rearrangements Drive Developmental Vessel Regression

**DOI:** 10.1371/journal.pbio.1002163

**Published:** 2015-05-14

**Authors:** Claudio A. Franco, Martin L. Jones, Miguel O. Bernabeu, Ilse Geudens, Thomas Mathivet, Andre Rosa, Felicia M. Lopes, Aida P. Lima, Anan Ragab, Russell T. Collins, Li-Kun Phng, Peter V. Coveney, Holger Gerhardt

An affiliation listed for the last author is incorrect. Holger Gerhardt is affiliated with the Vesalius Research Center (#6) rather than the University of Edinburgh (#5). The correct affiliations are as follows:

1. Vascular Biology Laboratory, London Research Institute—Cancer Research UK, Lincoln’s Inn Fields Laboratories, London, United Kingdom,

6. Vascular Patterning Laboratory, Vesalius Research Center, KU Leuven, Department of Oncology, VIB3, Leuven, Belgium

Current address: Max-Delbrück Center for Molecular Medicine, Berlin, Germany
