# Supplementary figures and images for: Dynamic Endothelial Cell Rearrangements Drive Developmental Vessel Regression
Source: PLoS Biol. 2015 Apr 17;13(4):e1002125. doi: 10.1371/journal.pbio.1002125 (PMC4401640; doi:10.1371/journal.pbio.1002125)

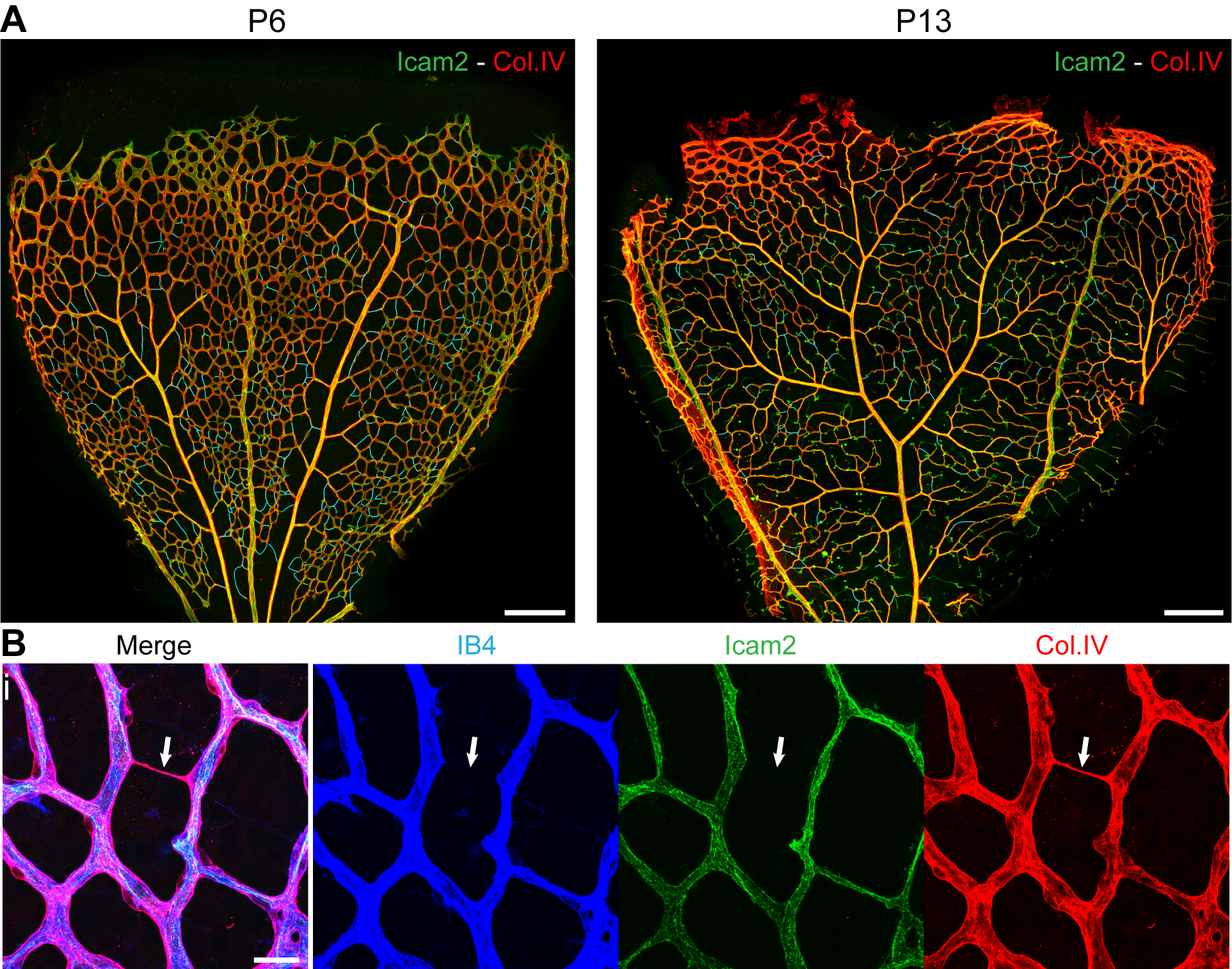

Supplement: S1 Fig — A, Overview of wild-type P6 and P13 mouse retinas highlighting all regression profiles (blue lines). Regression profiles are vessel segments with Col.IV-positive vessel segments and negative for ICAM2 staining or presenting a breakage in the continuity of the luminal staining. B, Typical basement membrane (Col.IV)-empty sleeve representing a regressed vessel segment (arrow) in a P6 retina. The basement membrane remains, while no lumenized vessel (ICAM2) or endothelial cell (IB4) is present. Scale bars (A: 200 μm; B: 20 μm). (TIF) [file pbio.1002125.s002.tif]

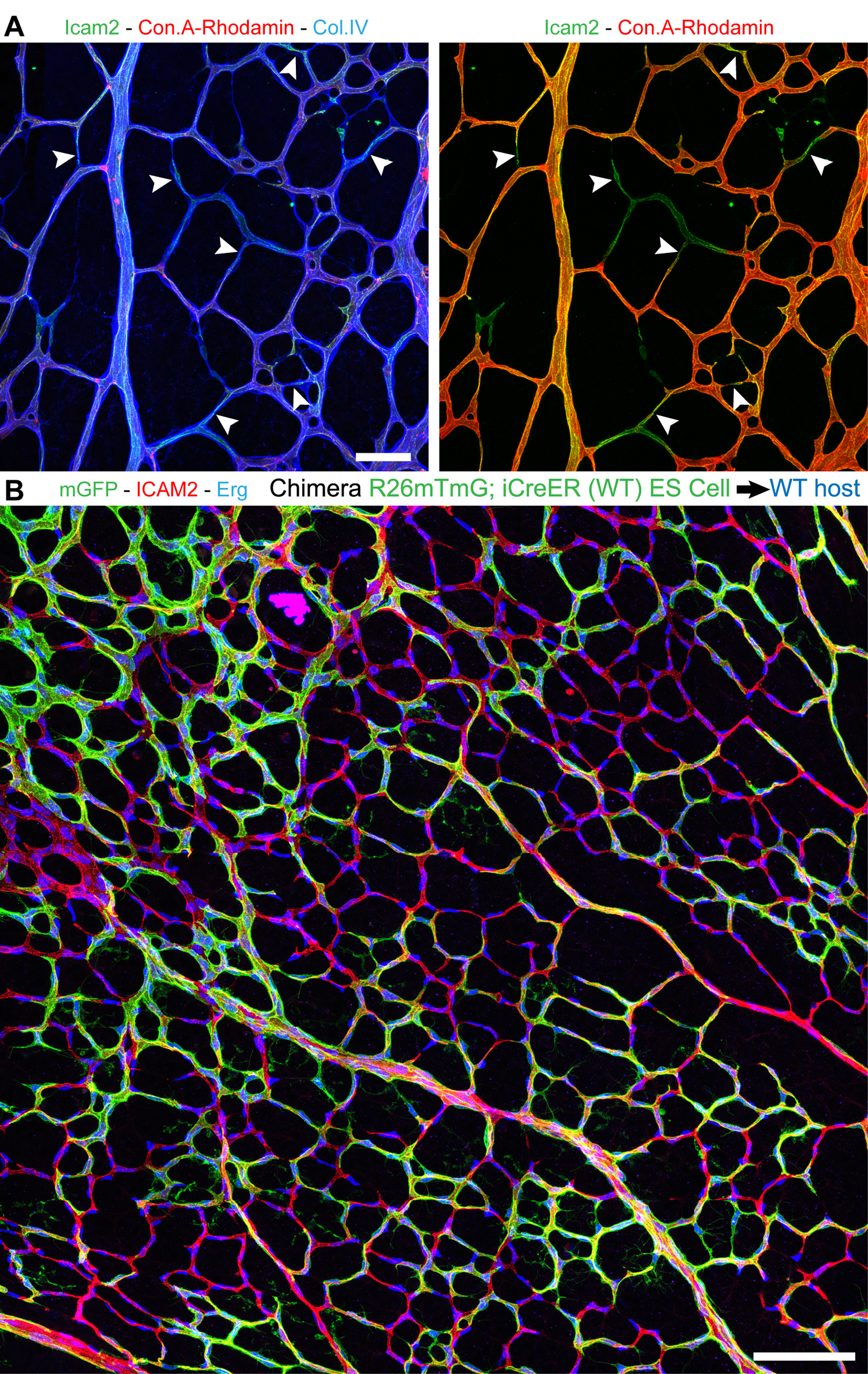

Supplement: S2 Fig — A, Confocal images of P6 wild-type retinas after fix-perfusion with rhodamin-conjugated concanavalin A (red). Retinas were stained for extracellular matrix (Col.IV) and the blood vessel lumen marker (ICAM2) and showed that regressing vessels correlated with rhodamin-negative vessel segments (arrows). B, Confocal images of chimeric retinas derived from injection of PDGFb-iCreER; R26mTmG ES cells into wild-type host blastocyst. Following tamoxifen-induced recombination at P2, retinas were collected at P6 and stained for endothelial nuclei (Erg) and a blood vessel lumen marker (ICAM2). Recombined endothelial cells (mGFP) were scattered throughout the vasculature and no-clonal expansion of endothelial cells in blood vessels could be seen. Scale bars (a: 50 μm; b: 200 μm). (TIF) [file pbio.1002125.s003.tif]

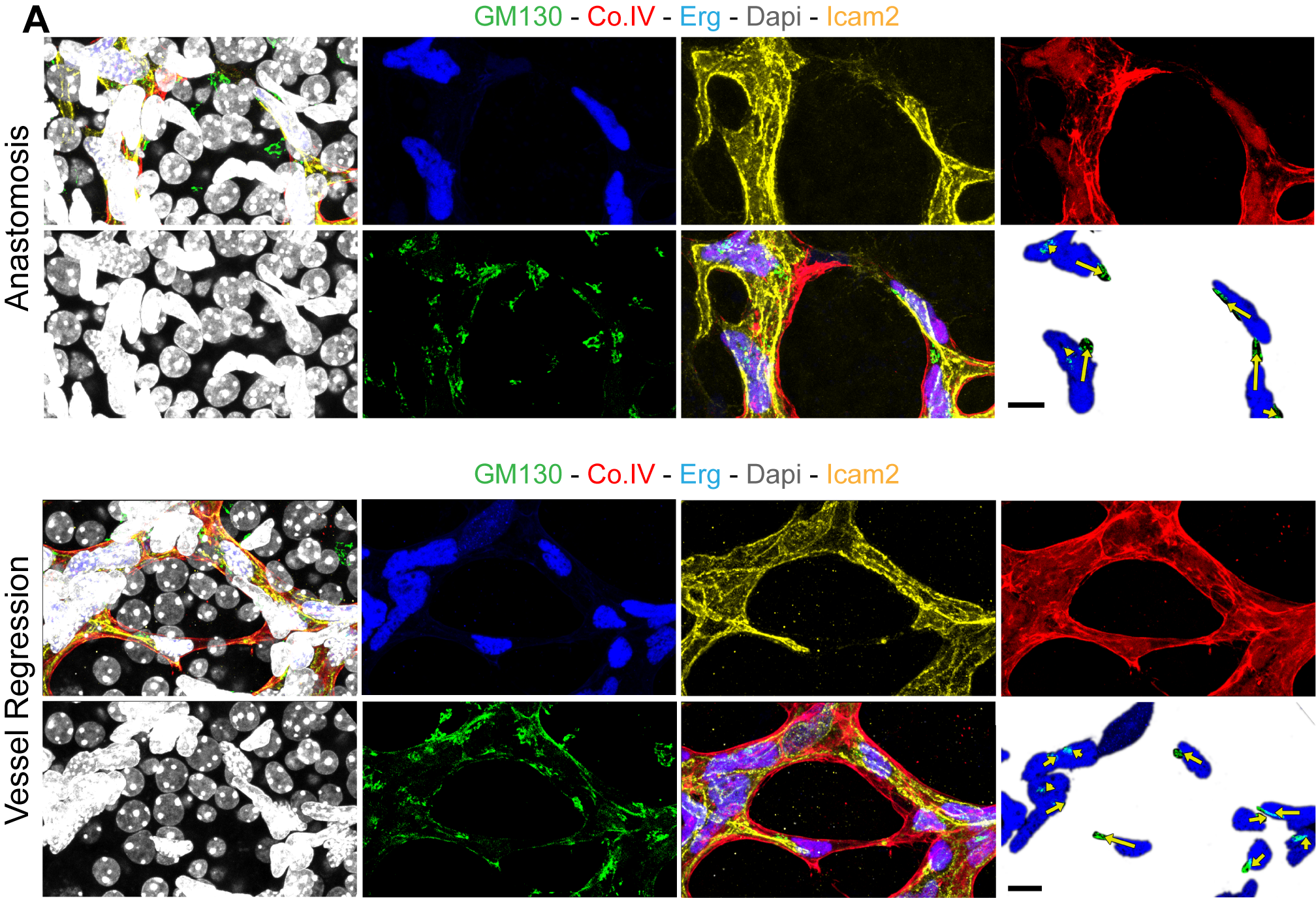

Supplement: S3 Fig — A, Non-treated image shown in Fig 3A, showing all the channels in separate panels. Labels for stainings are shown in figure. (TIF) [file pbio.1002125.s004.tif]

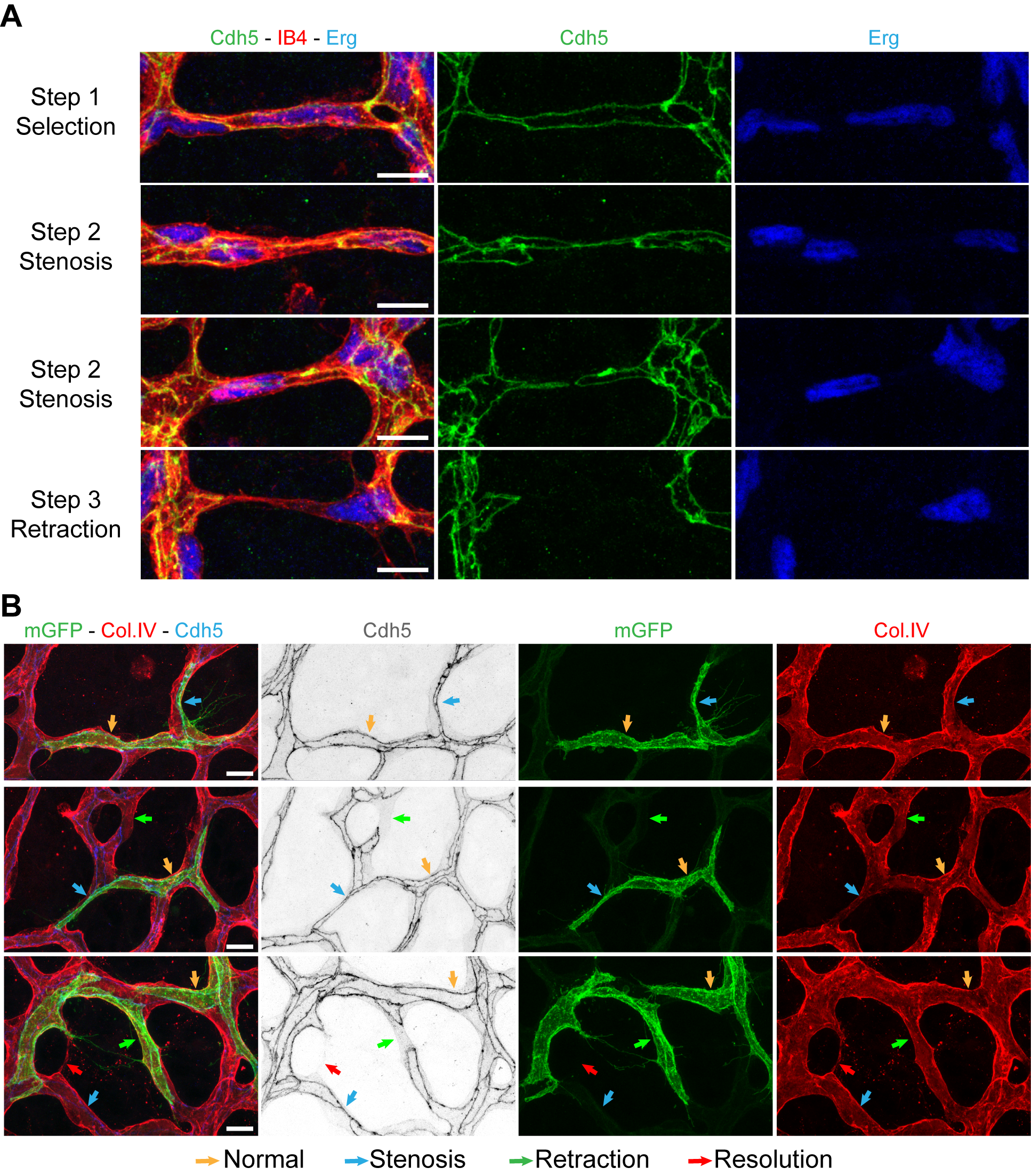

Supplement: S4 Fig — A, Confocal images of several vessel segments, stained markers for endothelial cells (IB4), junctions (Cdh5), and endothelial cell nuclei (Erg) in a wild-type P6 mouse retina. Vessel segments are categorized according to configurations described in Fig 4E. B, Single-endothelial cell labeling, using Cre-induced expression of membrane-bound GFP (mGFP), shows polarized morphology of endothelial cells in different stages of vessel regression, as defined by the color-coded arrows. Scale bars (A and B: 10 μm). (TIF) [file pbio.1002125.s005.tif]

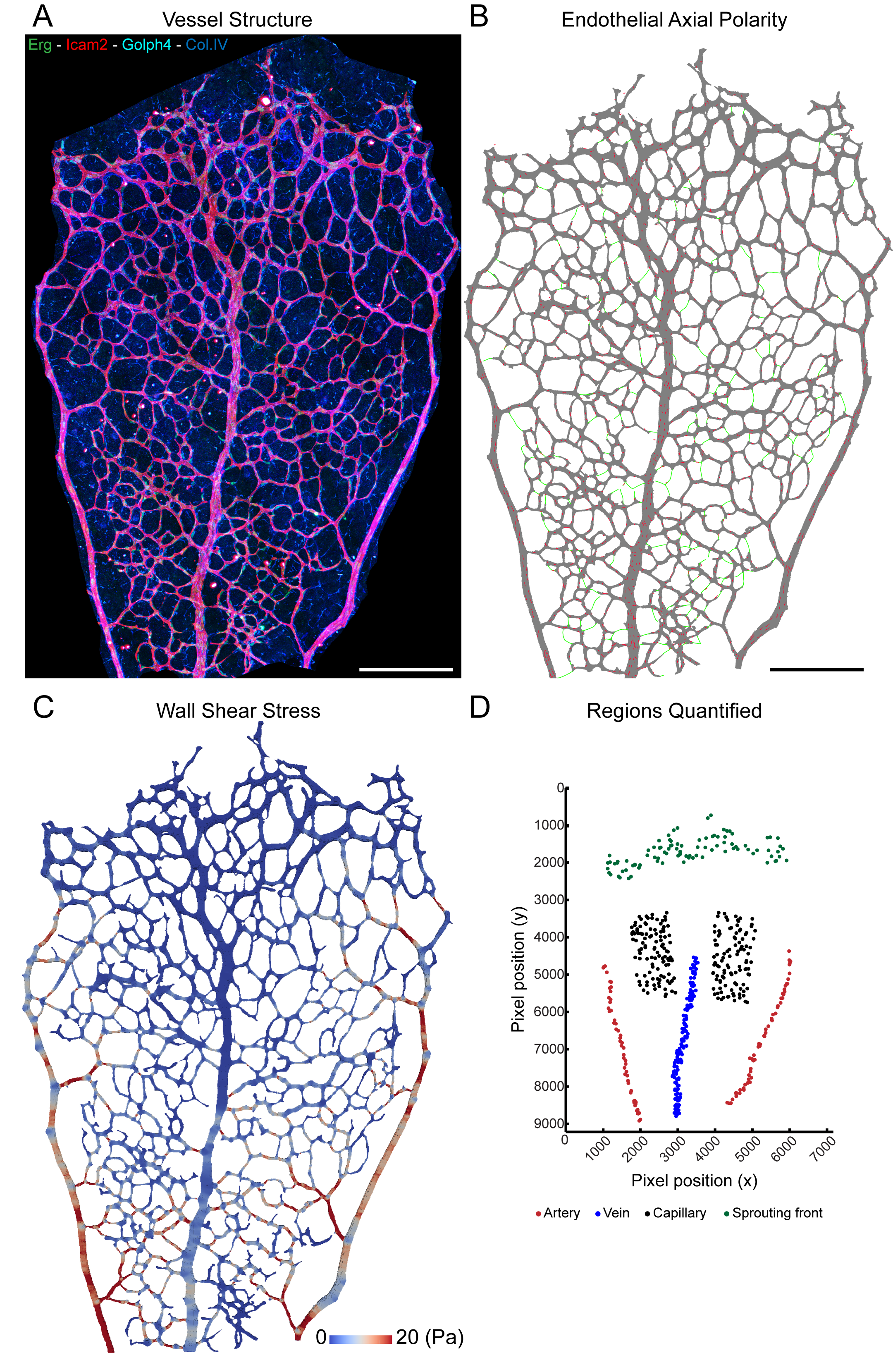

Supplement: S5 Fig — A, Wild-type P6 mouse retina stained for extracellular matrix (Col.IV), endothelial cell nuclei (Erg), blood vessel lumen (ICAM2) and Golgi apparatus (Golph4). B, Image segmentation of the vascular plexus of the mouse retina in (A), highlighting the lumen of blood vessels (grey), the regression profiles (green lines), and the nucleus-to-golgi (axial) polarity of all endothelial cells (red arrows). C, Color-coded shear stress map of in mouse retina vascular network in (A), predicted using a computational approach. D, Spatial representation of individual cells for each of the selected groups (artery, vein, capillary, and sprouting front) used for quantifications of axial polarity in Fig 5. Scale bars (A and B: 200 μm). (TIF) [file pbio.1002125.s006.tif]

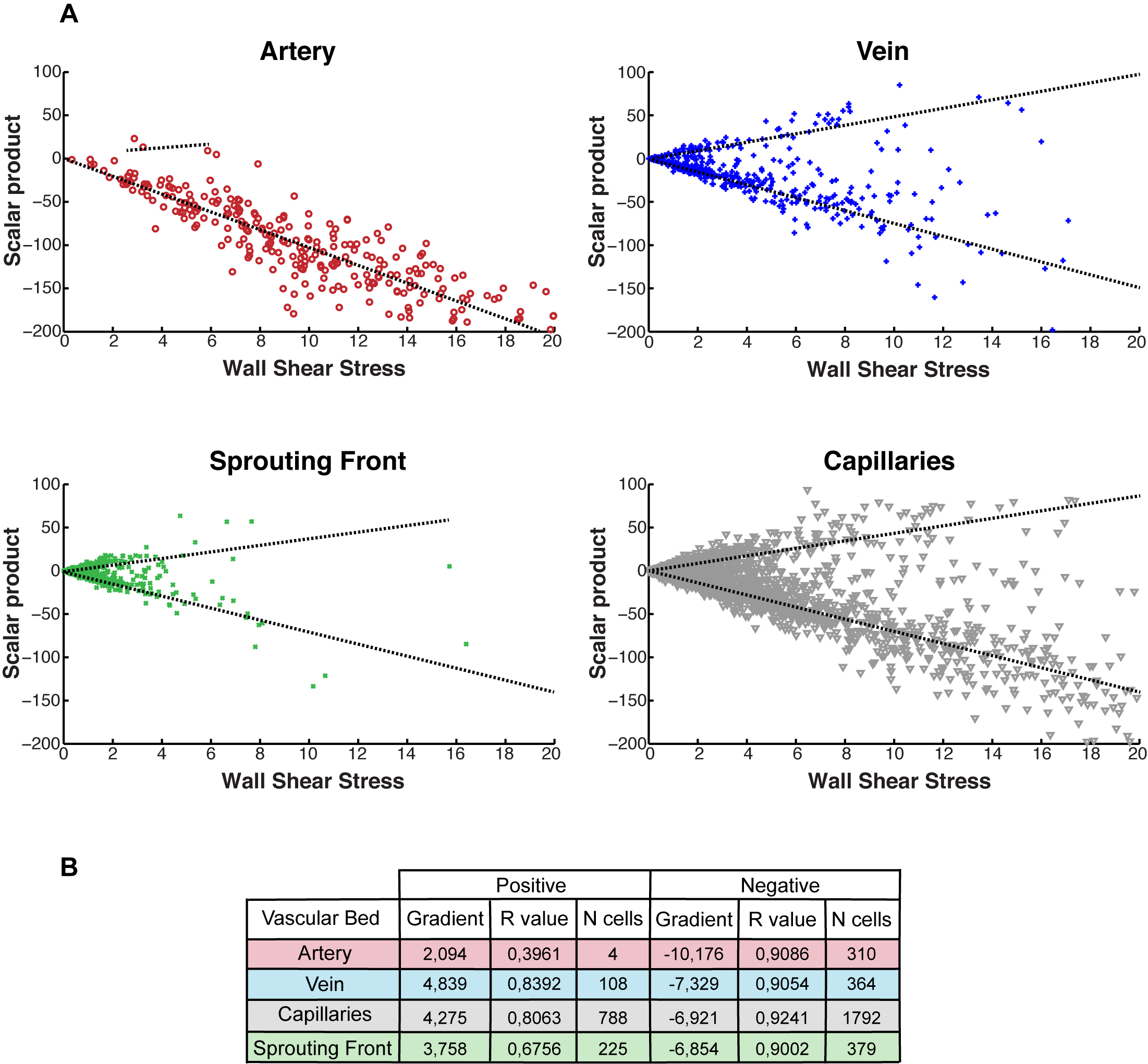

Supplement: S6 Fig — A, Graphs showing distribution of scalar products in function to wall shear stress levels for each vascular plexus. Scalar product corresponds to the product between length of the axial polarity vector and the cosine of the angle between the axial polarity vector and the flow direction vector. B, linear regression analysis of positive (polarized with flow) and negative (polarized against the flow) scalar product points for each endothelial cell nuclei. Gradient, R-value, and number of cells analyzed for each vascular bed is shown. n = 3 retinas. The data used to make this figure can be found in S1 Data. (TIF) [file pbio.1002125.s007.tif]
