# Supplementary material for: Dynamic Endothelial Cell Rearrangements Drive Developmental Vessel Regression
Source: PLoS Biol. 2015 Apr 17;13(4):e1002125. doi: 10.1371/journal.pbio.1002125 (PMC4401640; doi:10.1371/journal.pbio.1002125)
Supplement: S1 Table — (DOCX) [file pbio.1002125.s014.docx]

| **Antibody** | **Manufacturer** | **Cat. No.** | **Dilution** |
| --- | --- | --- | --- |
| Cleaved Caspase 3 | Cell Signaling | 9661 | 1:400 |
| Collagen IV | AbD Serotec | 2150-1470 | 1:400 |
| Concanavalin A-Rhodamin | Vector Labs | RL-1002 | 1:200 |
| Erg1/2/3 | Santa Cruz Antibodies | sc-353 | 1:200 |
| Golph4 | Abcam | Ab28049 | 1:400 |
| ICAM2 | BD Pharmingen | 553326 | 1:200 |
| Mouse VE-cadherin | BD Pharmingen | 555289 | 1:50 |
| ZO1 | Invitrogen | 40-2300 | 1:200 |
| Donkey a-Rabbit Alexa488 | Invitrogen | A-21206 | 1:400 |
| Donkey a-Rabbit Alexa647 | Invitrogen | A-31573 | 1:400 |
| Goat a-Rat Alexa555 | Invitrogen | A-21434 | 1:400 |
| Donkey a-Rat Alexa594 | Invitrogen | A-21209 | 1:400 |
| Donkey a-Goat Alexa647 | Invitrogen | A-21447 | 1:400 |
| Donkey a-Rabbit Fab fragment | Jackson’s Laboratories | 711-007-003 | 1:100 |
| IsolectinB4 – Alexa647 | Invitrogen | I-32450 | 1:500 |
